# Development of Forest Tree Species Composition: Selected Results of the National Forest Inventory of Lithuania

**DOI:** 10.3390/plants14050667

**Published:** 2025-02-21

**Authors:** Raimundas Petrokas, Michael Manton, Gintaras Kulbokas, Milda Muraškienė

**Affiliations:** 1 Department of Forest Genetics and Tree Breeding, Institute of Forestry, Lithuanian Research Centre for Agriculture and Forestry, Liepų Str. 1, LT-53101 Girionys, Lithuania; 2 Bioeconomy Research Institute, Vytautas Magnus University, Studentų Str. 13, LT-53362 Akademija, Lithuania; michael.manton@vdu.lt; 3 Lithuanian State Forest Service, Pramonės Str. 11A, LT-51327 Kaunas, Lithuania; gintaras.kulbokas@amvmt.lt; 4 Department of Silviculture and Ecology, Institute of Forestry, Lithuanian Research Centre for Agriculture and Forestry, Liepų Str. 1, LT-53101 Girionys, Lithuania; milda.muraskiene@lammc.lt

**Keywords:** closer to nature, forest types, forest management, forest inventory, forest development, tree species, stand age

## Abstract

Forest development forms the foundation for the advancement of sustainable forest management that integrates the knowledge of natural and anthropogenic processes with ecological and biological insights. This study aims to emphasize the role of assisted natural regeneration and balanced forest development phases in fostering closer-to-nature management approaches, contributing to resilient forest ecosystems capable of self-regulation and biodiversity support in the face of anthropogenic and climatic challenges. This study focuses on forest development in Lithuania based on five National Forest Inventories (NFIs) from 2002 to 2022. We examine the tree volume structure of the growing stock by stand type and forest type series from the point of view of stand age and forest development phases. This is performed by applying the standardized methodologies of the Lithuanian National Forest Inventory. Our analysis focuses on broader patterns derived from the selected NFI data rather than stand-level details. Our findings demonstrate that long-term observation of dynamic National Forest Inventories can aid in the development of closer-to-nature forest management methods for different forest type series. In order to implement the European Union’s strategy and policy for closer-to-nature forest management, we call for the use of “assisted succession” methods in commercial forests, promoting the formation of mixed-species forest stands with multi-cohort age profiles, including old-growth all-aged forest patches of >121 years.

## 1. Introduction

The European Union has been progressively developing its closer-to-nature forest management policy as part of its broader sustainability agenda [1]. This approach, stressed in the EU Forest Strategy for 2030 and the EU Biodiversity Strategy for 2030, aims to enhance biodiversity, improve forest resilience against climate change, and promote multifunctional forest ecosystems [2,3]. Closer-to-nature forest management seeks to integrate the ecological, social, and economic dimensions of forestry, by aligning with the principles of sustainable forest management (SFM) established in earlier EU policies [4].

The implementation of closer-to-nature forest management calls on each EU country to develop their own practices that involve adapting forestry practices to mimic natural processes, thereby fostering healthier ecosystems and enhancing carbon sequestration capabilities [5,6]. This paradigm shift is crucial as it addresses the building pressures on forest resources due to the rising demand for biomass and timber, while also mitigating biodiversity loss [7]. Furthermore, it provides opportunities for forest managers to maintain and re-create ecosystems that are not only productive but also resilient to climate change, thereby ensuring their long-term sustainability [8,9]. However, to achieve this requires the impartial understanding of natural forest patterns and processes, such as forest stand development, on the one hand, and the access to relevant forest data that can deliver current forest characteristics and trends, such as the National Forest Inventory (NFI) data, on the other [10,11].

Forest stand development and tree species composition are influenced by a range of factors, including ecological, climatic, and anthropogenic elements, which interact with the life history characteristics of the tree species [12,13]. Ecologically, the presence of shade-intolerant species is enhanced through practices like group selection, which allows for greater regeneration success by providing necessary light conditions [14]. The composition of undergrowth, particularly in fertile soils, indicates a transition from pioneer deciduous species to more stable deciduous hardwoods [15]. This can reflect both natural succession and management practices [16]. Climatic factors, i.e., temperature and precipitation patterns, also play a critical role in shaping species distribution and forest dynamics, as they affect growth rates and species interactions [17]. Natural disturbances such as windthrow, insects, disease, and fire can significantly alter forest stand structure and species composition, necessitating adaptive management strategies to maintain biodiversity and ecosystem health [18,19]. Furthermore, human activities, including forest management practices, land-use changes, drainage, and forest planting, disrupt natural processes, leading to shifts in species composition and forest integrity and resilience [20,21]. As climate change continues to alter the frequency and intensity of disturbances, it becomes increasingly important to understand the dynamics of forest stand development and to incorporate these dynamics into the management practices to ensure the sustainability of hemiboreal forests [18,22]. Thus, the understanding the life histories and adaptive strategies of tree species in response to competition, stress, and disturbance are vital for forest stand development and ecosystem resilience [2,9].

To capture the changes and trends in forest development and tree species composition as well as defining closer-to-nature forest management practices requires long-term monitoring, such as National Forest Inventories (NFIs) [23]. NFIs provide accurate, comprehensive, and up-to-date information data on forest resources, including tree species composition, age structure, biomass, and carbon stocks [24,25]. As climate change continues to pose significant challenges and opportunities for forest growth, the importance of NFIs in providing reliable data for closer-to-nature forest management strategies cannot be overstated. For example, NFI data can help identify changes in forest stand structure and composition over time, allowing for the evaluation of the impacts of both climate change and human activities on forest health [26,27]. Thus, NFIs are promising tools for assessing the health and dynamics of forest ecosystems and aiding in an informed decision-making process for closer-to-nature forest management.

Therefore, the aim of this study is to analyse the tree species compositions of hemiboreal forests in the context of forest development and sustainability, in order to support the transition to closer-to-nature forest management. To achieve this, we first examined the tree volume structure of the growing stock across different stand types and forest type series, as well as stand age and forest development phases, and second, we identified trends in forest composition, structural changes, and succession processes that influence long-term forest resilience. Finally, we discussed the future research needs of the sustainable management and evaluation of forest development to ensure that the physical and biological conditions of forest ecosystems self-regulate to support forest-dependent biodiversity.

## 2. Materials and Methods

### 2.1. Lithuania as a Case Study

We selected Lithuania, which lies completely within the European hemiboreal forest zone, as the case study. This forest zone forms a distinctive ecological region that serves as a transitional zone between boreal and temperate forests and is characterized by a rich diversity of flora and fauna, shaped by a combination of climatic conditions, soil types, and historical land use practices [9,28].

According to the Lithuanian Forestry Statistics [29], the area of forest land covers 33.7% of the country’s territory. Of this forest land, 74.9% is covered by commercial forests, in which the main objective of the economic activities is to form productive forest stands for a permanent timber supply. Strict reserve forests are distinguished in the forest land of the state only. Coniferous stands prevail in Lithuania, covering 55.7% of the forest area; they are followed by softwood deciduous forests, at 40.9%, while hardwood deciduous forests occupy 3.4% [29].

**Figure 1:**
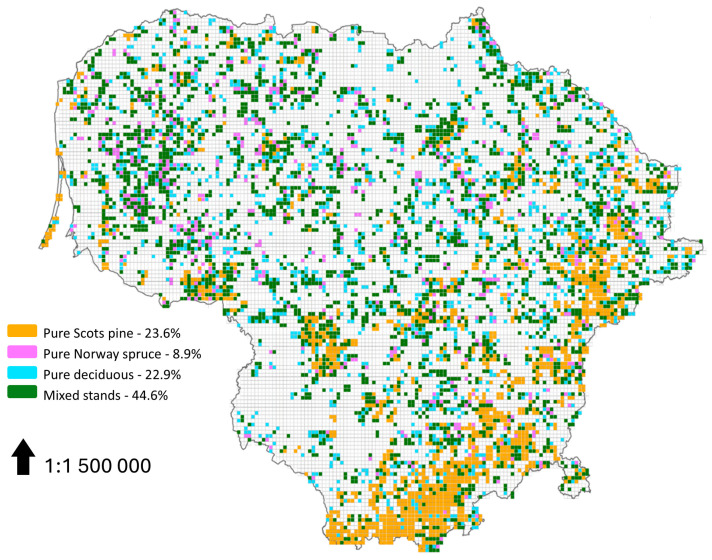
Forest stand species mixtures according to tree growing stock volume in Lithuania’s National Forest Inventory plots (N = 6113) for 2022. Pure stands are represented as dominant single species, containing >75%. Pure deciduous stands are represented by individual dominant tree species (except silver and downy birch, which were estimated together). Mixed stands include all possible tree species.

The mixture of Lithuanian forests is relatively stable, but the very obvious and constant tendencies of the increasing areas of softwood deciduous trees and decreasing areas of coniferous tree species cannot be ignored [30]. According to the Lithuanian NFI data, 55% of permanent forest inventory plots are single-species-dominated stands (i.e., >75%) based on growing stock volume (Figure 1). This can be accounted for by the dominant even-aged clearfell rotation system used throughout Lithuania’s forests [29,31].

Most of the changes in the tree species composition of Lithuanian forests are due to changes in the volume and age structure of timber harvests, depending on the growth of wood and the sanitary condition of the stands [32]. For instance, to prevent the natural loss of trees that die naturally due to competition, diseases, pests, or are destroyed by storms, snow, ice, drought, etc., the stands are thinned out or cut down in advance, and the target species composition is formed by taking high-quality living wood [30]. However, pre-harvesting high-quality timber or low-quality biodiversity trees does not guarantee a healthy forest. In Lithuania, the number of trees dying spontaneously or due to natural factors, pests, and diseases does not decrease (and makes up, on average, 17–19% of the gross volume increment), which shows the insufficient impact of silviculture measures on increasing the resilience and sustainability of the stands [30].

Thus, the long-term resilience of Lithuanian forests is increasingly challenged by climate change, pests, and human activities. Furthermore, the observed transitions in forest composition, such as the increasing share of Norway spruce (*Picea abies*), may lead to vulnerabilities, as Norway spruce-dominated forests are highly susceptible to drought, windthrow, and bark beetle infestations [33]. Ash dieback has been found to cause a large amount of damage, especially in humid forests where European ash (*Fraxinus excelsior*) grows densely [34,35]. Nevertheless, subsequent forest re-establishment practices with scarification and planting of singular tree species continues to decrease forest resilience in Lithuania. The situation is aggravated by the increase in the volume of logging in Lithuanian forests, which has doubled from 3.0 to 6.7 million cubic metres of timber between 1990 and 2020.

International processes individually emphasize some but not all the benefits from forests, with some variation over time [36]. Some current problems at the national level are related to poor links between information supply and demand. For example, in Lithuania, the need for discussion and information at the national level is currently due to climate change, biodiversity issues and carbon sequestration, while at the local level the emphasis is on wood supply. Furthermore, Lithuania’s current focus on forest area and area change provides a very poor evaluation of forest benefits, as most of them depend on other parameters. In conclusion, it should be noted that a national forest assessment should examine not only the biophysical resources of forests and trees, but also the sustainable management and use of these resources, with particular attention to ecosystem integrity [36,37].

### 2.2. Lithuanian National Forest Inventory

The Lithuanian National Forest Inventory (NFI) is a systematic approach that systematically collects, analyses, and reports data on the country’s forest resources, focusing on aspects like forest extent, composition, health, biomass, and biodiversity [30,38]. The main indicators that are analysed are the growing stock volume, volume increment, and stand density, overall and broken down by tree size. The widespread system of permanent forest sample plots (N = 6154) contains the geographical location of each tree, which ensures the identification of trees during subsequent inventories and delivers high data confidence [30]. According to the Lithuanian NFI, one permanent sample plot represents 400 ha of Lithuania’s forest area. The growing stock volume of all Lithuanian forest stands per 1 ha according to permanent sample plots is estimated with an accuracy of 1.0% (with a probability of 0.68). In all forests of Lithuania, the total growing stock volume is estimated with an error of 1.5%. This is especially important to objectively assess the impact of forest management activities on the formation of the stand [30].

Conducted at regular intervals, the Lithuanian NFI employs standardized methodologies to monitor and assess various parameters, such as tree species diversity, age classes, growth rates, and forest stand health. It uses tree-level inventory reporting, using objective systematic sampling with a random starting point. The main NFI sampling unit consists of a permanent circular plot, which consists of several elements for different measurement objectives (see Žemaitis et al. [39]). The main circular sample plot (500 m^2^ in size, horizontal radius equals 12.62 m) is used to measure all trees with a diameter greater than 14 cm at a height of 1.3 m. Basal area, species composition, age, and growing stock volume of the stand are assessed throughout the stand in a horizontal radius of 12.62 m, and the forest type series, site type, as well as soil type in a horizontal radius of 30 m [39,40]. Wood resources in Lithuanian forests are expressed in terms of tree stem volume, in cubic meters (solid) per unit area (1 ha) or in the total forest stand area [41,42,43].

This study analysed Lithuanian NFI data from five 5-year remeasurement cycles spanning 1998–2002 (NFI 2002), 2003–2007 (NFI 2007), 2008–2012 (NFI 2012), 2013–2017 (NFI 2017), and 2018–2022 (NFI 2022), covering all regions of Lithuania. Using this dataset, we examined growing stock volume, volume increment, stand density, tree distribution, and growing stock characteristics by diameter classes and age profiles. A tree volume stratification model was employed to explore how biodiversity mechanisms known to enhance ecosystem biomass production are linked to forest development [44,45]. Standardized methodologies of the Lithuanian NFI were applied; the volume structure of the growing stock (I and II storey) was determined using species-specific indices. Individual tree species’ stem volumes were further converted into relative values following Lithuania’s NFI standards [41].

The study of the relationship between tree age structure and species composition across different stand types and forest type series is an important area of our current and future research. It focuses on broader patterns derived from the selected NFI data rather than stand-level details. While stand-level data, such as the proportion of real monocultures, even-aged, and uneven-aged stands, would indeed provide valuable insights, such data were beyond the scope of our current study due to the structure of the available NFI dataset.

### 2.3. Forest Vegetation and Soil Dynamics

Forest development is simply the secondary succession or the orderly and predictable change in the dominant species of forest plants [46]. It is jointly determined by multiple factors pertaining to three dimensions, i.e., climatic, edaphic, and biotic [47]. The mosaic of natural hemiboreal forest communities is largely the result of ecological succession leading ultimately to climatic climax vegetation [14]. In some parts of the hemiboreal forest zone, there are areas where local peculiarities of the soil or topography make the development of the climatic climax forest impossible, so the potential natural forest communities here are edaphic climaxes on immature sand soils or biotic climaxes on hydromorphic soils associated with marshes, swamps, or poorly drained uplands [21].

Multiple edaphic factors influence different aspects of the hemiboreal forest stand structure, including different tree life stages and species composition. The distribution, richness, and structure of forest species are related to both edaphic and topographic characteristics; the spatial dependence of forest–plant diversity on soil diversity indicates the integrity of the community with the bedrock [48,49]. Soils whose fertility is determined by bedrock are less diverse, while soils that are not associated with bedrock in their mineral composition are more diverse. Soil fertility differentiates the ways in which interactions between plants and bedrock develop [48]. Forest species composition is primarily influenced by the type of bedrock (especially its base saturation), soil reaction, and nutrients [50]. If there are no differences in base saturation, then the second most important variables are those related to the terrain (especially slope), which affect the availability of light and moisture. For example, peat thickness is strongly related to topographical features at macroscales, which can explain more than 40% of the peat thickness spatial variability [51].

Forest ecosystems create soil heterogeneity through organisms, litter, and disturbance. However, modern soil classification uses soil morphology aligned with parent material, climate, and time, and assumes continuous soil formation at landscape and millennial scales [52]. In mainstream forestry (age-class management with pure stands), the different classifications of forest development and algorithms used to assign a forest patch to a specific forest development phase does not take into account soil dynamics at the temporal and spatial scales of trees and stand structures [52,53,54]. However, soil series can be useful when combined with other characteristics to delineate stands. The Lithuanian NFI does not include the Food and Agriculture Organization (FAO) soil classification system but continues to assess site types based on soil typological groups [55,56]. To be consistent with FAO approach, we have classified the main forest habitats of Lithuania into forest type series, site types, and soil types (Table 1), including 13 Natura 2000 forest habitat types of European Community importance [57,58,59]. Table 1 was used to select the NFI data, which were further analysed in the results. Nevertheless, since the Lithuanian NFI data on soil typological groups are based on the old soil classification system, our study does not include an analysis of forest soils.

### 2.4. Forest Development

In this study, the phases of secondary forest development are characterized using a general scheme of patch development [62]. Although there is no consensus among scientists on how many phases a forest life cycle has [53], we applied a general classification of forest development phases (Table 2) that defined the phases of stand initiation, stem exclusion, understorey reinitiation, and steady state [46,63]. The stand initiation phase starts following the cessation of the disturbance [46]. An example of such a disturbance is clear-cutting, which resets the successional process to the beginning. Stand initiating disturbances are sufficiently intense and widespread that large amounts of sunlight are available at or near the soil of the forest floor. The early successional dominants, such as Scots pine (*Pinus sylvestris*), silver and downy birch (*Betula pendula* and *Betula pubescens*), Eurasian aspen (*Populus tremula*), and grey and black alder (*Alnus incana* and *Alnus glutinosa*), form the early canopy. The duration of this phase is the shortest, perhaps 15 to 25 years. The stem exclusion phase starts as soon as the tree vegetation forms a closed canopy. For many pioneer species in this phase, self-thinning takes place, allowing sunlight to penetrate deeper and eventually promoting the development of post-pioneer species in the understorey, in other words, the establishment of the understorey. Due to the difference in the moment of death of individual trees, the release of the post-pioneer understorey also occurs differently [62]. For fast-growing species, this phase can last 20 to 30 years. Meanwhile, the duration of the understorey reinitiation phase is highly dependent on the longevity of the dominant species. For example, the lifespan of early dominant Eurasian aspen and silver birch can be 60 to 100 years, whereas if Scots pine and English oak (*Quercus robur*) are dominant, the lifespan might be well over 120 years. The steady state phase represents the mature forest. The early successional dominants may have died out; the forest regenerates itself among longer-lived “pre-climax” trees, such as European white elm (*Ulmus laevis*), wych elm (*Ulmus glabra*), small-leaved lime (*Tilia cordata*), Norway maple (*Acer platanoides*), and European beech (*Fagus sylvatica*), which can survive in the sunlight available through small openings in the canopy as single trees die. In a forest development context, this final phase occurs when all trees in the forest are “climax” species established in the shade of other trees [64].

Forests that have been recently killed or altered by fire, insects, disease, wind or logging are still forests because of the biological and physical legacies from the previous forest [65]. Under a regime of sustainable forest management, many or most of these legacies persist during the period between forest disturbance and the redevelopment of tree cover. Therefore, to ensure the resilience and sustainability of the stands, it is important to retain structural forest legacies and apply assisted natural regeneration using a variety of species [14]. Natural reforestation and artificial reforestation are two distinct approaches to forest restoration. Natural reforestation relies on the spontaneous regeneration of native species through natural processes, often influenced by existing seed banks and surrounding vegetation, leading to diverse ecosystems [66,67]. In contrast, artificial reforestation involves human intervention, typically through planting specific tree species, which may not always replicate the ecological complexity of natural forests [68,69]. Whereas natural afforestation occurs when native species colonize non-forest land through land abandonment, artificial afforestation involves the deliberate planting of trees in these areas [70]. Both methods aim to enhance biodiversity and ecosystem services, but their ecological outcomes can differ significantly due to the methods employed and species selected [71].

All potential trees should be provided the opportunities to develop throughout the four phases (see Table 2) to continue delivering forest resilience and legacy [62]. Therefore, we examined the tree volume structure of the growing stock by stand type and forest type series from the point of view of the stand age and forest development phases. The initial step in comprehending stand successional pathways under various disturbance regimes is age structure [72]. Indirect evidence of stand-replacing disturbances can be seen in even-aged stands. Following partial cohort-replacing disturbances, uneven-aged stands form; the severity of the disturbance is indicated by the ratio of younger to older tree cohorts. In the absence of major disturbances, all-aged stands develop over several centuries of succession.

In a coupled human–nature system, where local adaptations guide the ecosystem toward local and global optimums, anthropogenic modification can be detected by the distributions of correlated patches [37,73]. However, it was not possible to achieve this in our study. A stand-wise forest inventory in Lithuania is carried out by recording the actual condition of the stands, so the full history of thinning and fellings under the established forest management regime is not visible [74]. Thus, we aimed to identify unsustainable forestry practices that need immediate attention [75].

**Table 2:** A conceptual ecological framework for the development phases of the hemiboreal forest. The background colour indicates the development phase characteristic of the successional status of a group of tree species. Tree species are listed in descending order of light need [14].

Potential Natural Forest Vegetation (Forest Type Series) *	Forest Development Phases: Successional Status of Tree Species			
	Stand Initiation (0–20 Years): Pioneer	Stem Exclusion and Understorey Initiation (20–60 Years): Post-Pioneer	Understorey Reinitiation (60–120 Years): Pre-Climax	Steady State (>120 Years): Climax
Climatic (*aeg cmh hox mox ox oxn*)	*Pinus sylvestris* (*hox mox ox*) *Betula pubescens* (*aeg cmh oxn*) *Betula pendula* (*aeg cmh hox mox ox oxn*) *Salix fragilis* (*aeg*) *Populus tremula* (*aeg cmh hox mox ox oxn*) *Alnus incana* (*aeg cmh hox oxn*) *Alnus glutinosa* (*aeg cmh oxn*)	*Pinus sylvestris* (*hox mox ox*) *Betula pubescens* (*aeg cmh oxn*) *Betula pendula* (*aeg cmh hox mox ox oxn*) *Salix fragilis* (*aeg*) *Populus tremula* (*aeg cmh hox mox ox oxn*) *Alnus incana* (*aeg cmh hox oxn*) *Alnus glutinosa* (*aeg cmh oxn*) *Quercus robur* (*aeg cmh hox mox ox oxn*) *Fraxinus excelsior* (*aeg cmh oxn*) *Carpinus betulus* (*aeg hox*) *Picea abies* (*aeg hox mox ox oxn*)	*Pinus sylvestris* (*hox mox ox*) *Betula pendula* (*aeg cmh hox mox ox oxn*) *Alnus glutinosa* (*aeg cmh oxn*) *Quercus robur* (*aeg cmh hox mox ox oxn*) *Fraxinus excelsior* (*aeg cmh oxn*) *Carpinus betulus* (*aeg hox*) *Picea abies* (*aeg hox mox ox oxn*) *Ulmus glabra* (*aeg cmh hox oxn*) *Ulmus laevis* (*aeg cmh hox*) *Tilia cordata* (*aeg cmh hox oxn*) *Acer platanoides* (*aeg cmh hox ox oxn*) *Fagus sylvatica* (*hox*)	*Pinus sylvestris* (*hox mox ox*) *Quercus robur* (*aeg cmh hox mox ox oxn*) *Fraxinus excelsior* (*aeg cmh oxn*) *Carpinus betulus* (*aeg hox*) *Picea abies* (*aeg hox mox ox oxn*) *Ulmus glabra* (*aeg cmh hox oxn*) *Ulmus laevis* (*aeg cmh hox*) *Tilia cordata* (*aeg cmh hox oxn*) *Acer platanoides* (*aeg cmh hox ox oxn*) *Fagus sylvatica* (*hox*)
Edaphic (*cal cl fil m ur v vm*)	*Pinus sylvestris* (*v cl vm m*) *Betula pubescens* (*cal fil ur*) *Betula pendula* (*cal fil m ur v vm*) *Salix fragilis* (*ur*) *Salix alba* (*ur*) *Populus tremula* (*m vm*) *Alnus incana* (*fil*) *Alnus glutinosa* (*cal fil ur*)	*Pinus sylvestris* (*v cl vm m*) *Betula pubescens* (*cal fil ur*) *Betula pendula* (*cal fil m ur v vm*) *Salix fragilis* (*ur*) *Salix alba* (*ur*) *Populus tremula* (*m vm*) *Alnus incana* (*fil*) *Alnus glutinosa* (*cal fil ur*) *Quercus robur* (*cal*) *Fraxinus excelsior* (*fil ur*) *Carpinus betulus* (*ox*) *Picea abies* (*cal fil m ur vm*)	*Pinus sylvestris* (*v cl vm m*) *Betula pendula* (*cal fil m ur v vm*) *Alnus glutinosa* (*cal fil ur*) *Quercus robur* (*cal*) *Fraxinus excelsior* (*fil ur*) *Carpinus betulus* (*ox*) *Picea abies* (*cal fil m ur vm*)	*Pinus sylvestris* (*cl m v vm*) *Quercus robur* (*cal*) *Fraxinus excelsior* (*fil ur*) *Carpinus betulus* (*ox*) *Picea abies* (*cal fil m ur vm*)
Biotic (*c cir csp lsp msp*)	*Pinus sylvestris* (*csp lsp msp*) *Betula pubescens* (*c cir csp msp*) *Alnus glutinosa* (*c cir*)	*Pinus sylvestris* (*csp lsp msp*) *Betula pubescens* (*c cir csp msp*) *Alnus glutinosa* (*c cir*) *Picea abies* (*c cir*)	*Pinus sylvestris* (*csp lsp msp*) *Alnus glutinosa* (*c cir*) *Picea abies* (*c cir*)	*Pinus sylvestris* (*csp lsp msp*) *Picea abies* (*c cir*)

* Forest type series (ground layer codes) are indicated in brackets [76]: *aeg—Aegopodiosa*, *c—Caricosa*, *cal—Calamagrostidosa*, *cir—Carico-iridosa*, *cl—Cladoniosa*, *cmh—Carico-mixtoherbosa*, *csp—Carico-sphagnosa*, *fil—Filipendulo-mixtoherbosa*, *hox—Hepatico-oxalidosa*, *lsp—Ledo-sphagnosa*, *m—Myrtillosa*, *mox—Myrtillo-oxalidosa*, *msp—Myrtillo-sphagnosa*, *ox—Oxalidosa*, *oxn—Oxalido-nemorosa*, *ur—Urticosa*, *v—Vacciniosa*, *vm—Vaccinio-myrtillosa*.

## 3. Results

### 3.1. Growing Stock Volume of Lithuanian Forest Stands

**Figure 2:**
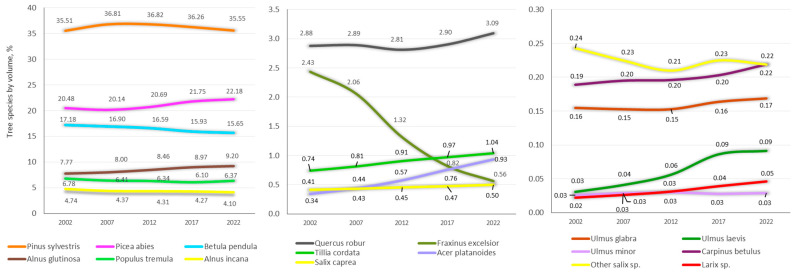
Three levels of tree species biodiversity in Lithuanian forests according to the National Forest Inventory data from five 5-year remeasurement cycles. Numbers show the percentages of the growing stock volume of the tree species from the growing stock volume of all tree species: (1) more than 3%, (2) 0.3–3%, and (3) 0.03–0.3%.

Currently, the largest share of the volume of tree stems is made up of Scots pine and Norway spruce (Table 3, Figure 2), which are considered the most valuable tree species. There is a different distribution between the stem volumes of individual tree species in mature stands, taking into account the participation of each tree species in the species composition of stands of other dominant tree species [30]. According to the Lithuanian NFI data for 2022, the percentage of growing stock volume of predominant tree species from the growing stock volume of all tree species is 82% for Scots pine and 74% for Norway spruce stands. Scots pine is the least common admixture in forest stands of other dominant tree species. Stands of silver and downy birch, as well as Eurasian aspen are mostly mixed stands, where the volume of the predominant species is 62%, as are English oak (57%) and European ash (44%) stands. The most widespread admixture in forest stands of other tree species is Norway spruce.

The changes in tree species composition by volume in Lithuania from 2002 to 2022 showed that Scots pine and Norway spruce exhibit relatively stable trends, with Scots pine consistently maintaining the highest share (around 35%), whereas Norway spruce has increased from 20.14% to 22.18% (Figure 2). Silver and downy birch and black alder display slight declines over the study period. Minor increases are observed in grey alder and Eurasian aspen, while less common species like English oak and small-leaved lime exhibit a gradual increase. In contrast, European ash has significantly declined from 2.43% in 2003 to 0.56% in 2022. Changes in the other species, such as such as Norway maple, crack and white willow (*Salix fragilis* and *Salix alba*), and European hornbeam (*Carpinus betulus*), were negligible. Overall, these trends indicate a stable dominance of a few species, with slight shifts in diversity towards the less abundant tree species.

**Figure 3:**
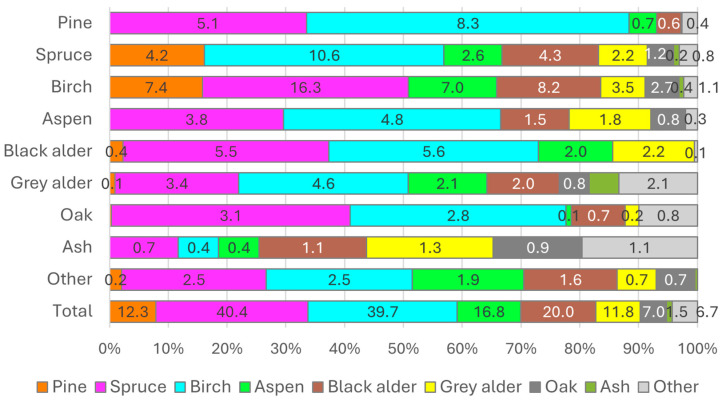
Natural change in forest stand type over 5 years (2017–2022), excluding clear-cut areas. The label shows the change area per 1000 ha.

The natural change in forest stand types in Lithuania over the last 5-year period (2017–2022) showed that the most significant transitions occurred in mixed stands, where species like silver and downy birch (16.3%), Norway spruce (10.6%), and black alder (5.6%) contributed substantially to the changes (Figure 3). Scots pine stands decreased significantly, transitioning towards Norway spruce (8.3%) and silver and downy birch (5.1%) shifts. Silver and downy birch stands showed large transitions to other species, ranging from 16.3% for Norway spruce to 7.4% and 7.0% towards Scots pine and Eurasian aspen, respectively. The other less dominant tree species accounted for minor contributions, reflecting their lower prevalence. Overall, silver and downy birch and Norway spruce were the primary species driving natural stand changes, while Scots pine showed consistent replacement trends across the analysed period. This highlights the dynamic nature of forest composition in response to environmental and successional processes.

### 3.2. Growing Stock Composition and Structure of Lithuanian Forest Stands

The tree volume structure of the growing stock (I and II storey) was studied by stand type and forest type series, depending on the stand age and forest development phases (see Table 2). According to the Lithuanian NFI, the proportion of Norway spruce growing stock in stands of Scots pine, silver and downy birch, Eurasian aspen, and black alder is increasing with age (Table 4). In early successional phases (0–60 years), silver birch and downy birch are the main secondary species in all other tree stands. Norway spruce is the main companion in stands of other tree species at the phases of understorey reinitiation and steady state (≥61 years). European ash stands do not follow the general pattern of forest development.

Silver and downy birch stands are found in almost all series of forest types. Mixed Norway spruce forests (i.e., *Oxalidosa*, *Myrtillo-oxalidosa*, *Hepatico-oxalidosa*, and *Oxalido-nemorosa* forest type series) have stands of all tree species (Table 4). The predominant stand type in the *Oxalidosa* series was found to be Scots pine with Norway spruce (Table 5); however, it is well known that the main forest type in this series is the *Oxalidosa-Piceetum* with Scots pine and English oak [21]. European ash stands have survived in only one series of forest types, the *Aegopodiosa*. It is necessary to note that there is no mature or old-growth forest in the *Aegopodiosa*, *Carico-mixtoherbosa*, and *Urticosa* series of forest types (see Table 5). However, it is well known that the main forest types in these series (i.e., *Aegopodio-Quercetum*/*Fraxinetum*, *Carico-mixtoherbo-Fraxinetum*/*Quercetum*, and *Urtico-Alnetum glutinosae/Fraxinetum*) are dominated by long-living “post-pioneer” English oaks and European ashes [21].

The analysis of the tree volume structure of growing stock across various forest types and development phases, as indicated by the Lithuanian National Forest Inventory data for 2022, reveals distinct patterns in species prevalence (Table 5). The *Aegopodiosa* forest type exhibited the highest dominance of a single species (English oak, 100% for the youngest cohort), while the *Vacciniosa* type showed remarkable stability with near-complete dominance across all age classes with Scots pine. Forest types such as *Myrtillo-oxalidosa* and *Oxalidosa* displayed notable variability in species (silver birch, Norway spruce, Scots pine, and English oak) composition across different age classes, suggesting a dynamic growth structure. In contrast, the *Cladoniosa* lacked sufficient data for analysis. Overall, the findings highlight the diverse ecological characteristics of Lithuanian forests, with variations in tree species volume distribution influenced by both age and forest type.

### 3.3. Stand Regeneration

Comparing species stand regeneration composition and area changes of forest regeneration over the past 20 years showed a variation between natural reforestation (NR), artificial reforestation (AR), natural afforestation (NA), and artificial afforestation (AA). NR predominantly features black alder (34.4 thousand ha) and grey alder (24.1 thousand ha), Eurasian aspen (43.7 thousand ha), and Norway spruce (13.9 thousand ha). AR is dominated by Norway spruce (102.6 thousand ha), followed by Scots pine (32.9 thousand ha), and black alder (16.8 thousand ha), accounting for the most substantial contribution to forest recovery. For NA, grey alder(19.1 thousand ha), Scots pine (17.6 thousand ha), and black alder(10.5 thousand ha) play leading roles, with lesser contributions from Eurasian aspen. AA showed a smaller overall area, with Norway spruce (12.1 thousand ha) and Scots pine (6.8 thousand ha) as the primary species.

**Figure 4:**
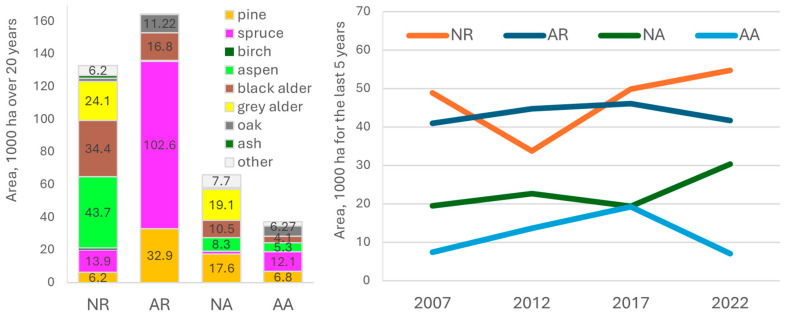
The comparison of species composition and changes in area of natural and artificial reforestation/afforestation over the last 20 years. NR—natural reforestation, AR—artificial reforestation, NA—natural afforestation, and AA—artificial afforestation.

The temporal trends (2007–2022) revealed a steady area increase in NR, particularly after 2017, while AR fluctuates but remains significant. NA demonstrates moderate growth, whereas AA has declined. This indicates that artificial interventions, particularly in reforestation, have been crucial for large-scale forest recovery, while natural processes are increasingly contributing to afforestation (see Figure 4).

## 4. Discussion and Conclusions

### 4.1. Implications of Current Forest Management

The results highlight a stable dominance of Scots pine and Norway spruce, accounting for significant shares of the growing stock volume—82% and 74%, respectively. However, there is a gradual increase in less common species such as English oak, small-leaved lime, and grey alder, alongside a concerning decline in European ash. These trends underscore the limited diversity of dominant forest stand species in Lithuania and the vulnerability of certain tree species to environmental or biotic stressors.

The natural transition of forest stands between 2017 and 2022, particularly the replacement of Scots pine with Norway spruce and silver birch, reflect the dynamic nature of forest ecosystems. These changes are influenced by successional processes, competition, environmental conditions, loss of natural processes (e.g., [13]), and a push for faster-growing timber [77]. However, the dominance of a few species like Norway spruce in the later phases of forest development (≥61 years) suggests a lack of structural diversity in premature stands [78,79]. The increase in grey alder and Eurasian aspen could be due to increased harvesting as well as land abandonment, which leads to the formation of a younger forest age profile (0–60 years), since both tree species are pioneers and do not tolerate shade (see Figure 4).

Lithuania’s forests, dominated by Scots pine and Norway spruce, provide high timber value but may compromise other ecosystem services, such as biodiversity support, and soil protection [80]. Early successional species like silver and downy birch are critical for soil stabilization and nutrient cycling, while long-lived species like English oak provide habitats for diverse fauna and flora [81]. The decline of European ash, a keystone species in certain deciduous forest ecosystems, further highlights the need to balance timber production with ecological considerations [82].

Although the individual results of the Lithuanian NFI, presented for stand types, forest type series, and age classes, show good tree species diversity for all units studied, the absence of mature and old-growth stands in certain forest type series, such as *Aegopodiosa*, *Carico-mixtoherbosa*, and *Urticosa*, further highlights the gaps in forest management strategies. Old-growth all-aged forests serve as benchmarks for natural ecosystem functions [83]; they provide a variety of niches and habitats, supporting a higher number of plant, fungus, and animal species [84]. Unfortunately, the Lithuanian NFI does not analyse the actual condition of the old-growth all-aged forests. Moreover, in Lithuanian forestry, old-growth stands are considered overmature and require renewal [85]. Although the locations of permanent NFI sample plots are not published and their locations are kept secret, foresters may actively protect them and thus promote over- and underestimations. For instance, NFI data showed a significant increase in the number of large trees greater than 50 cm in diameter from 2.9 to 5.5 trees/ha^−1^ (https://amvmt.lrv.lt/lt/naujienos/ar-stambiu-medziu-miskuose-mazeja/ (accessed on 23 January 2025)). Nevertheless, Mozgeris et al. [86] forecast that the availability of large trees for large nesting birds will be decreased, thus indicating a loss in old large trees. So, EU policy requires consolidation in Lithuanian forest legislation. Currently, the final felling of natural forest habitats of European Community importance is halted until national forest management laws are amended, in line with the EC. However, the NFI still provides a valid dataset for broad national trends.

In summary, it can be said that Lithuanian forestry is focused on the wood resources of healthy stands using clear-cut forestry that reduces tree species age diversity, by locally favouring dominant tree species and typically restricting harvesting age to less than 121 years. In Lithuania, strict nature reserves are the only areas where forests have the potential to turn into ancient woods; as of now, they make up only 1.3% of Lithuanian forest territory and are located on nonproductive peatlands [29,87]. However, according to the NMI data, almost 10% of the forest area in Lithuania is classified as natural forest habitats of EC importance and must have closer-to-nature forest management practices developed.

### 4.2. A Case for Closer-to-Nature Forest Management

Paradigm shifts are required in traditional European forestry practices to reconcile human activities with the preservation of natural ecosystems [88]. Traditional forest management has prioritized economic outputs, leading to practices that diminish biodiversity and disrupt ecological natural patterns and processes. For instance, timber-oriented management has been linked to declining populations of forest species, [20,89]. This conflict between anthropogenic interests and ecological integrity emphasizes the need for a more integrated approach to forest governance that incorporates ecological, social, and economic dimensions [5,90]. Recent studies advocate for sustainable multifunctional forest management, which emphasizes the importance of diverse management practices that enhance ecosystem resilience and biodiversity [91]. The current trajectory of forest management in Europe, characterized by a historical focus on maximizing timber yield, is unsustainable and poses the risk of a “war with nature”, where ecosystems are pushed beyond their capacity to recover [92,93]. Therefore, a shift towards governance models that prioritize ecological health, such as those outlined in the pan-European criteria for sustainable forest management, is imperative [94]. This shift aims to mitigate the adverse impacts of human activities on forest ecosystems and seeks to foster a harmonious coexistence between human needs and the natural environment, thereby reducing the inevitability of conflict with nature.

All sustainable forest management requirements must include the maintenance, conservation, and enhancement of ecosystem biodiversity, the protection of ecologically important forest areas, and the prohibition of forest conversions [95,96]. Closer-to-nature forest management, with its emphasis on mixed-species stand structures that can be found in forests under natural dynamics, can address the implications of current forestry by fostering biologically integrated communities together with the local soils and climates with which they interact [1,65]. Mixed stands are more resilient to pests, diseases, and climate change, providing a buffer against catastrophic losses and maintaining ecosystem functions [97]. For instance, promoting the integration of deciduous broadleaved trees in appropriate habitats can help restore their populations while enhancing structural complexity and biodiversity [98]. This would also lead towards a dynamic forest composition and adaptability of forest ecosystems that prioritizes the emulation of natural processes, allowing for more gradual and adaptive stand transitions [99]. This approach encourages the development of multilayered canopies, uneven-aged stands, and natural regeneration, which aligns closely with Lithuania’s observed complex dynamics of forest ecosystems [14]. For instance, allowing silver and downy birch, Norway spruce, and other species to regenerate together can create more resilient and self-sustaining forests.

Another aspect of closer-to-nature management is to enhance the delivery of ecosystem services. This can be achieved through the maintenance of forest-dependent species that fulfil key ecological roles [100]. Practices such as selective thinning, natural and assisted regeneration, and the preservation of deadwood can enhance key ecosystem functions such as water regulation and habitat provision, contributing to the multifunctionality of forests [3]. However, there may be trade-offs between the benefits that less-modified forests offer (such as regulatory functions) and those production services that require some anthropogenic modification (e.g., timber production) [37]. Overall, forests largely free of significant modification, i.e., forests having high ecosystem integrity, typically provide higher levels of many benefits than modified forests of the same type [101].

Closer-to-nature management can also address the need for a diverse forest age structure by extending rotation periods, applying different harvesting techniques, and prioritizing the restoration of mature and old-growth characteristics in appropriate forest types [102]. This includes retaining legacy trees and promoting long-lived native species and long-term naturally developing stand structures to recreate the ecological integrity of natural forests [37,74,103]. Modern management still aims towards single species and age stand profiles which lack variation, whilst natural forming stand communities are generally comprised mixed species with various ontogenetic age dynamics [14]. According to the Lithuanian NFI [30], the implementation of the sustainable forest management principle means the constant renewal of stands as the main precondition for maintaining a stable wood increment. The natural development of forest patches can be fostered through targeted management activities, in particular by carrying out intermediate felling aimed at maintaining a natural light balance within multiple canopies in order to achieve a mosaic of near-natural forming stand communities of different ontogenetic age profiles and, as a result, a more sustainable forest use [14,30,73,104].

To promote the implementation of more nature-friendly forest management methods, forest soil assessment should become an integral part of long-term monitoring, such as national forest inventories. Evaluating soil typology can help identify more sustainable forestry practices. Generally, forest soils can lose their resilience through large-scale homogenization of forests and soils via salvage logging, agriculture-like soil preparation, and seedling planting in plantation forestry [52]. For instance, previous studies [105,106] have indicated that sandy soils are particularly sensitive to intensive forest harvesting due to their low capacity to retain organic matter.

Finally, closer-to-nature management helps mitigate climate and anthropogenic pressures by fostering forest-dependent biodiversity and habitat heterogeneity [107]. Many species benefit from increased tree diversity, which also boosts other essential ecosystem services including productivity and disease and pest resistance [108]. Incorporating broadleaves and pioneer species into conifer-dominated forests enhances their resilience to environmental stressors [109]. Adaptive management, guided by long-term monitoring such as the National Forest Inventory can ensure that commercial forests remain robust and capable of withstanding future uncertainties [110].

## 5. Final Remarks

In conclusion, the stand development of European hemiboreal forests is a complex interplay of natural and anthropogenic factors. The integration of nature-based forestry practices into EU forest policy represents a significant step towards sustainable forest management, addressing the challenges posed by climate change and biodiversity loss. This study showed that National Forest Inventories serve as a vital tool in this context, providing essential data for informed decision-making and adaptive management.

Using data from the Lithuanian NFI, this study showed that more than half of Lithuanian forest stands are dominated by single tree species; Scots pine (24%), Norway spruce (9%), and individual deciduous species (23%), applying a 75% dominant single-species threshold limit. This highlights the need for a paradigm shift in forest management. Nature-based forestry can enhance forest-dependent biodiversity, long-term resilience, and ecosystem services while maintaining timber production. By emulating natural processes, diversifying native species, and prioritizing ecological integrity, Lithuania can create productive, self-sustaining forests. This transition requires policy support, stakeholder engagement, and investment in research and monitoring. The long-term ecological and economic benefits make it essential for Lithuania’s forests. Sustainable management should focus on “assisted succession”, respecting the ontogenetic age dynamics of stand-forming tree species and diversifying harvest ages. Letting nature lead is a core principle of sustainable forest management.

## Figures and Tables

**Table 1:** The typology of the hemiboreal forests of Lithuania.

Forest Habitats (NATURA 2000)	Forest Type Series *	Site Types **	Soil Types ****
Scots pine forests (9010 9060 91D0 91T0)	*Cladoniosa* (*cl*)	Šae, Nae	AR, RG
	*Vacciniosa* (*v*)	Šal, Nal	AR, PZ, RG
	*Vaccinio-myrtillosa* (*vm*)	Šbl, Šbp, Nbl, Nbp	AR, PZ
	*Myrtillosa* (*m*)	Lal, Lbl, Lbp	AR, PL, PZ
	*Myrtillo-sphagnosa* (*msp msps*)	Ual, Ubl, Ubp, Ma, Mb ***	GL, PZ
	*Carico-sphagnosa* (*csp csps*)	Pb, Mb	HSf-s
	*Ledo-sphagnosa* (*lsp lsps*)	Pa, Ma	HSf
Mixed Norway spruce forests (9050 9160 9180 9190 9070)	*Oxalidosa* (*ox*)	Šcl, Šcp, Šcs, Ncl, Ncp, Ncs	AR, LV, RT, PL, CM, FL
	*Myrtillo-oxalidosa* (*mox*)	Lcl, Lcp, Lcs	AR, LV, RT, PL, CM
	*Hepatico-oxalidosa* (*hox*)	Šdl, Šdp, Šds, Ndl, Ndp, Nds	AR, LV, RT, PL, CM, FL
	*Oxalido-nemorosa* (*oxn*)	Ldl, Ldp, Lds	LV, CM, FL
Mixed broadleaved forests (9020 9080 91F0 91E0)	*Aegopodiosa* (*aeg*)	Šfp, Šfs, Nfp, Nfs	LV, CM
	*Carico-mixtoherbosa* (*cmh*)	Lfl, Lfp, Lfs	LV, CM
	*Calamagrostidosa* (*cal cals*)	Ucl, Ucp, Ucs, Mc ***	GL
	*Filipendulo-mixtoherbosa* (*fil fils*)	Udl, Udp, Uds, Md ***	GL, FL
	*Urticosa* (*ur*)	Ufl, Ufp, Ufs	GL
	*Carico-iridosa* (*cir cirs*)	Pd, Md	HSs
	*Caricosa* (*c cs*)	Pc, Mc	HSs

* Dominant ground vegetation types: *s* in the ground layer code is the index of drained (*siccatum*) forest sites. ** Hydrotope codes in Lithuania: Š and N—slopes and normal water regime; L—temporarily overmoistured; U—overmoistured; P—peatland, thickness of peat layer: Pa, Pb ≥ 60 cm; Pc, Pd ≥ 40 cm; M—drained peatland. Trophotope and soil structure codes in Lithuania: a—very oligotrophic, b—oligotrophic, c—mesotrophic, d—eutrophic, f—very eutrophic, e—degraded, l—light soils sand–gravel, s—heavy soils clays, and p—light soil on heavy soil [60]. *** Thickness of peat layer in mineral soils: Ma, Mb, Mc, Md—up to 40 cm [61]. **** RT—Retisols, AR—Arenosols, CM—Cambisols, FL—Fluvisols, GL—Gleysols, HSf—Fibric Histosols, HSf-s—Terri-Fibric Histosols, HSs—Pachiterric Histosols, LV—Luvisols, PL—Planosols, PZ—Podzols, and RG—Regosols [56,57,61].

**Table 3:** Tree volume structure of Lithuanian forest stands according to the National Forest Inventory data from five five-year remeasurement cycles. The numbers show the percentage of growing stock volume of the tree species designated by the letter from the growing stock volume of all tree species. The bold text in brackets is the stand type area percentage **(%)**.

Stand Type *	NFI 2022	NFI 2017	NFI 2012	NFI 2007	NFI 2002
Scots pine (p)	82p 10e 5b **(31.5)**	83p 10e 5b **(32.0)**	83p 10e 5b **(32.9)**	84p 9e 5b **(33.5)**	84p 9e 5b **(34.1)**
Norway spruce (e)	74e 8b 6p **(18.6)**	74e 8b 7p **(17.8)**	73e 9b 7p **(17.5)**	72e 9b 7p **(18.2)**	71e 10b 7p **(17.8)**
Silver and downy birch (b)	62b 15e 5j **(19.9)**	62b 15e 6j **(20.3)**	62b 15e 6j **(20.5)**	61b 14e 6j **(20.3)**	61b 14e 6j **(19.6)**
Eurasian aspen (d)	62d 12e 10b **(7.3)**	62d 12e 11b **(6.9)**	61d 13e 11b **(6.7)**	60d 13e 12b **(6.4)**	60d 13e 12b **(6.3)**
Black alder (j)	72j 11b 7e **(11.3)**	71j 12b 8e **(11.1)**	70j 13b 8e **(10.2)**	69j 13b 8e **(9.3)**	67j 13b 8e **(9.1)**
Grey alder (bt)	70bt 8b 5e **(6.3)**	71bt 8b 6e **(6.4)**	73bt 8b 5e **(6.6)**	74bt 8b 4e **(6.4)**	73bt 9b 5e **(6.9)**
English oak (a)	57a 14e 8b **(2.6)**	56a 14e 8b **(2.5)**	55a 16e 7b **(2.4)**	55a 16e 7b **(2.2)**	56a 14e 8b **(2.4)**
European ash (u)	44u 16k 9e **(0.5)**	51u 10k 10e **(0.7)**	58u 7e 6d **(1.2)**	59u 7e 7d **(2.1)**	61u 7e 7d **(2.4)**
Other species	26l 17k 7bl **(2.1)**	27l 14k 7b **(2.3)**	28l 13k 8bl **(2.0)**	27l 12k 10a **(1.5)**	22l 11k 10bl **(1.5)**
All stands	36p 22e 16b **(100)**	36p 22e 16b **(100)**	37p 21e 17b **(100)**	37p 20e 17b **(100)**	36p 20e 17b **(100)**

* Tree species in descending order of light need (ID): p—*Pinus sylvestris*, b—*Betula pubescens* and *Betula pendula*, d—*Populus tremula*, bt—*Alnus incana*, j—*Alnus glutinosa*, a—*Quercus robur*, u—*Fraxinus excelsior*, e—*Picea abies*, g—*Ulmus glabra*, l—*Tilia cordata*, and k—*Acer platanoides* [14].

**Table 4:** Tree volume structure of the growing stock (I and II storey) by stand type according to the Lithuanian NFI data for 2022. The numbers show the percentage of the volume of the dominant tree species and the subsequent two tree species. The appointed letter refers to the tree species.

Stand Type *		Forest Development Phases/Stand Age (Years) **						Forest Type Series ***
	≤20	21–40	41–60	61–80	81–100	101–120	≥121	
Scots pine (p)	88p	85p	86p	84p	80p	79p	80p	*cal cals cl cmh csp csps hox lsp lsps m mox msp msps ox oxn v vm*
	4b	7b	6b	8e	14e	14e	15e	
	4e	5e	5e	5b	4b	4b	3b	
Norway spruce (e)	47e	77e	81e	73e	71e	66e	64e	*c cs cal cals cir cirs cmh csp csps fil fils hox m mox msp msps ox oxn vm*
	13a	8b	8b	8b	8p	11p	13p	
	12p	4a	3p	8p	8b	9b	7b	
Silver and downy birch (b)	60b	69b	67b	59b	54b	49b	-	*aeg c cs cal cals cir cirs cmh csp csps fil fils hox lsp lsps m mox msp msps ox oxn v vm*
	9e	10e	11e	19e	21e	29e		
	9p	5j	5j	6d	7j	7j		
Eurasian aspen (d)	65d	64d	60d	62d	60d	-	-	*aeg c cs cal cals cir cirs cmh csp csps fil fils hox mox ox oxn ur*
	11b	10b	13b	16e	20e			
	9e	7e	12e	9b	10b			
Black alder (j)	71j	74j	78j	69j	57j	60j	62j	*c cs cal cals cir cirs cmh csp csps fil fils hox mox ox oxn ur urs*
	10b	13b	10b	13b	19e	27e	36e	
	8e	4e	4e	9e	11b	10b	2a	
Grey alder (bt)	62bt	72bt	71bt	69bt	-	-	-	*aeg c cs cal cals cir cirs cmh fil fils hox mox ox oxn*
	14b	8b	8b	14e				
	5j	5e	5j	5j				
English oak (a)	48a	43a	57a	58a	56a	58a	56a	*aeg c cs cmh fil fils hox mox ox oxn*
	18b	28b	17e	17e	13e	9e	16e	
	17l	11e	11b	11b	10b	8l	8l	
European ash (u)	48u	47gl	46k	60u	48u	46u	44e	*aeg c cs cir cirs fil fils hox mox ox oxn*
	24e	35u	33u	13k	20l	38a	32u	
	10bt	8bt	8a	10a	13b	13b	14b	
Other species	18a	20bl	22l	35l	54l	30k	75l	*aeg c cs cal cals cmh fil fils hox mox ox oxn ur urs*
	16gl	12gl	13k	23k	14k	27l	25e	
	13bl	10k	10gl	8a	7u	14sb		

* Tree species (ID): p—*Pinus sylvestris*, e—*Picea abies*, b—*Betula pendula* and *Betula pubescens*, d—*Populus tremula*, j—*Alnus glutinosa*, bt—*Alnus incana*, a—*Quercus robur*, u—*Fraxinus excelsior*, k—*Acer platanoides*, l—*Tilia cordata*, bl—*Salix caprea*, gl—*Salix fragilis* and *Salix alba*, and sb—*Carpinus betulus*. ** Stand initiation (0–20 years), stem exclusion and understorey initiation (20–60 years), understorey reinitiation (60–120 years), and steady state (>120 years). *** *aeg—Aegopodiosa, c—Caricosa, cal—Calamagrostidosa, cir—Carico-iridosa, cl—Cladoniosa, cmh—Carico-mixtoherbosa, csp—Carico-sphagnosa, fil—Filipendulo-mixtoherbosa, hox—Hepatico-oxalidosa, lsp—Ledo-sphagnosa, m—Myrtillosa, mox—Myrtillo-oxalidosa, msp—Myrtillo-sphagnosa, ox—Oxalidosa, oxn—Oxalido-nemorosa, ur—Urticosa, v—Vacciniosa, and vm—Vaccinio-myrtillosa* (*s*—index of drained (*siccatum*) forest types; the underlined series of forest types includes stands of all tree species).

**Table 5:** Tree volume structure of the growing stock (I and II storey) by forest type series according to the Lithuanian NFI data for 2022. The numbers show the percentage of the volume of the dominant tree species and the subsequent two tree species. The appointed letter refers to the tree species.

Forest Type Series *	Forest Development Phases/Stand Age (Years) **							NFI 2022
	≤20	21–40	41–60	61–80	81–100	101–120	≥121	
*Cladoniosa* (*cl*)	NED	NED	NED	NED	NED	NED	NED	NED
*Vacciniosa* (*v*)	100p	78p	97p	98p	98p	99p	99p	97p
		17b	3b	2b	1b	1pk	1b	2b
		2pb			1pk			
*Vaccinio-myrtillosa* (*vm*)	77p	66p	78p	87p	85p	84p	87p	84p
	12b	18e	12e	8e	12e	13e	10e	11e
	9e	14b	9b	4b	3b	3b	2b	5b
*Myrtillosa* (*m*)	45p	53e	49e	56p	59p	69p	66p	55p
	30e	24b	31p	30e	31e	25e	27e	32e
	18b	21p	9b	12b	9b	5b	5b	10b
*Myrtillo-sphagnosa* (*msp msps*)	37p	44b	39p	65p	62p	53p	64p	54p
	35b	35e	33b	27e	18e	37e	29e	27e
	7a	17p	28e	7b	15b	9b	6b	17b
*Carico-sphagnosa* (*csp csps*)	71b	55b	43b	50p	65p	64p	69p	53p
	10p	16d	25p	23b	19e	25e	21e	22b
	8e	13p	19e	20e	14b	10b	7b	19e
*Ledo-sphagnosa* (*lsp lsps*)	57p	67b	80p	88p	94p	91p	95p	82p
	43b	27p	17b	12b	4b	5e	4b	15b
		4j	3e		1d	4b		2e
*Oxalidosa* (*ox*)	28b	31e	43p	51p	47p	56p	59p	44p
	20e	21b	21e	22e	32e	23e	22e	24e
	14d	19p	17b	13b	9b	8b	11a	14b
*Myrtillo-oxalidosa* (*mox*)	28b	39e	39e	41e	54e	47e	44e	42e
	22e	23b	23b	22b	16p	22p	36p	20b
	15d	10bt	10j	14p	12b	14b	13a	11p
*Hepatico-oxalidosa* (*hox*)	23b	26d	19e	17b	30e	29e	29e	21e
	18a	22bt	14bt	16e	19b	24a	24a	14b
	18bt	18e	13b	15p	15a	13l	23p	13d
*Oxalido-nemorosa* (*oxn*)	26d	25e	30e	27b	35b	33e	40a	26e
	17b	20bt	19bt	25e	23e	19b	25e	22b
	14bt	18b	17b	20d	13d	12a	8d	16d
*Aegopodiosa* (*aeg*)	100a ***	66bt	36bt	34u	38d	47b	-	25bt
		11bl	25e	24b	32a	32a		15u
		8b	17u	23bt	14k	10e		14b
*Carico-mixtoherbosa* (*cmh*)	23b	26bt	32b	42b	22e	36l	-	25b
	17e	24e	23bt	26d	17a	35b		17bt
	17bt	19d	15j	11j	13d	21a		17d
*Calamagrostidosa* (*cal cals*)	53j	36b	37j	36j	30b	31e	62e	35j
	25b	32j	31b	30e	21e	26b	18b	28b
	8e	15e	20e	19b	21j	25j	15d	22e
*Filipendulo-mixtoherbosa* (*fil fils*)	60j	38j	56j	51j	37j	37e	53e	48j
	14b	19b	22b	20b	27b	28b	30u	21b
	11bt	13bt	8bt	11e	22e	26j	10b	11e
*Urticosa* (*ur*)	68j	32gl	29bt	82j	41j	-	-	53j
	22u	29j	27d	8b	40b			13b
	10b	19b	16j	5bt	8u			10bt
*Carico-iridosa* (*cir cirs*)	90j	37j	89j	62j	45j	76j	-	71j
	9b	24b	7b	27b	32e	22e		15b
	1a	17e	3e	10e	24b	3b		10e
*Caricosa* (*c cs*)	60j	50j	51j	33j	30b	39e	-	42j
	18b	35b	31b	31b	28e	22j		31b
	7p	5e	8e	20e	27j	19b		14e

* Scots pine forests: *cl*, *v*, *vm*, *m*, *msp*, *msps*, *csp*, *csps*, *lsp*, and *lsps*; mixed Norway spruce forests: *ox*, *mox*, *hox*, and *oxn*; mixed broadleaved forests: *aeg*, *cmh*, *cal*, *cals*, *fil*, *fils*, *ur*, *cir*, *cirs*, *c*, and *cs*; *s*—index of drained (*siccatum*) forest types. ** Stand initiation (0–20 years), stem exclusion and understorey initiation (20–60 years), understorey reinitiation (60–120 years), and steady state (>120 years). *** Tree species (ID): p—*Pinus sylvestris*, pb—*Pinus banksiana*, pk—*Pinus mugo*, e—*Picea abies*, b—*Betula pendula* and *Betula pubescens*, d—*Populus tremula*, j—*Alnus glutinosa*, bt—*Alnus incana*, a—*Quercus robur*, u—*Fraxinus excelsior*, k—*Acer platanoides*, l—*Tilia cordata*, bl—*Salix caprea*, and gl—*Salix fragilis* and *Salix alba*. NED—Not Enough Data.

## Data Availability

Data were obtained from the Lithuanian State Forest Service. Requests to access the datasets should be directed to Gintaras Kulbokas, Lithuanian State Forest Service.
